# Heat Shock Proteins HSPA1 and HSP90AA1 Are Upregulated in Colorectal Polyps and Can Be Targeted in Cancer Cells by Anti-Inflammatory Oxicams with Arylpiperazine Pharmacophore and Benzoyl Moiety Substitutions at Thiazine Ring

**DOI:** 10.3390/biom11111588

**Published:** 2021-10-27

**Authors:** Izabela Szczuka, Jarosław Wierzbicki, Paweł Serek, Berenika M. Szczęśniak-Sięga, Małgorzata Krzystek-Korpacka

**Affiliations:** 1 Department of Biochemistry and Immunochemistry, Wroclaw Medical University, 50-368 Wroclaw, Poland; izabela.szczuka@umed.wroc.pl (I.S.); pawel.serek@umed.wroc.pl (P.S.); 2 Department of Minimally Invasive Surgery and Proctology, Wroclaw Medical University, 50-556 Wroclaw, Poland; jaroslaw.wierzbicki@umed.wroc.pl; 3 Department of Medicinal Chemistry, Faculty of Pharmacy, Wroclaw Medical University, 50-556 Wroclaw, Poland; berenika.szczesniak-siega@umed.wroc.pl

**Keywords:** drug repurposing, colorectal cancer, chemoprevention, nonsteroidal anti-inflammatory drugs, piroxicam, meloxicam, molecular-targeted therapies, colorectal adenomas, proteostasis

## Abstract

Heat shock proteins HSPA1/Hsp70α and HSP90AA1/Hsp90α are crucial for cancer growth but their expression pattern in colorectal polyps or whether they can be modulated by oxicams is unknown. We quantified (RTqPCR) *HSPA1* and *HSP90AA1* expression in 50 polyp-normal pairs in relation to polyp malignancy potential and examined the effect of piroxicam, meloxicam and five novel analogues on HSPA1 and HSP90AA1 expression (mRNA/protein) in colorectal adenocarcinoma lines. *HSPA1* and *HSP90AA1* were upregulated in polyps by 3- and 2.9-fold. Expression ratios were higher in polyps with higher dysplasia grade and dominant villous growth pattern, mostly a result of diminished gene expression in normal tissue. Classic oxicams had negligible/non-significant effect on HSP expression. Their most effective analogue inhibited HSPA1 protein and gene by 2.5-fold and 5.7-fold in Caco-2 and by 11.5-fold and 6.8-fold in HCT116 and HSPA1 protein in HT-29 by 1.9-fold. It downregulated HSP90AA1 protein and gene by 1.9-fold and 3.7-fold in Caco-2 and by 2-fold and 5.0-fold in HCT116. *HSPA1* and *HSP90AA1* are upregulated in colorectal polyps reflecting their potential for malignancy. HSPA1 in cancer cells and, to lesser degree, HSP90AA1 can be reduced by oxicam analogues with thiazine ring substituted via propylene linker by arylpiperazine pharmacophore with fluorine substituents and by benzoyl moiety.

## 1. Introduction

Recent years have been associated with the rapid development of molecularly-targeted therapies. In colorectal cancer (CRC), they are principally addressed to patients with gross metastatic disease or primary tumors non-amenable for curative resection. For these patients, chemotherapy is a leading treatment option, although it is unsatisfactorily efficient, inducing resistance and characterized by high systemic toxicity. Potential molecular targets are researched from among pathways crucial for cancer growth, survival and progression [1]. Cancer-associated inflammation and its mediators fulfil these criteria as sustaining inflammation is included among key cancer characteristics, nurturing functionality of other hallmarks [2]. Consistently, a number of anti-inflammatory drugs, including oxicams—a class of nonsteroidal anti-inflammatory drugs (NSAIDs), has displayed chemopreventive and/or anti-tumor activities (reviewed in [3,4,5]), also in CRC [6]. As such, oxicams have been approved as non-cancer drugs with anti-tumor activity and listed in the Repurposing Drugs in Oncology (ReDO) database [7]. 

Structurally, oxicams are distinguished by lack of carboxyl group characterizing most of NSAIDs. Like other NSAIDs, they inhibit synthesis of prostanoids by interfering with cyclooxygenase (COX) activity. However, they can bind to a separate pocket in the enzyme and also inhibit microsomal prostaglandin E synthase-1 (mPGES-1), a major player in prostaglandin E2 synthesis during inflammatory response [8]. Anti-neoplastic properties of piroxicam and meloxicam—oxicam representatives—have been repeatedly demonstrated [9,10,11,12,13,14,15,16] and are known to be exerted by both COX-dependent and independent routes [14]. Still, the exact molecular mechanisms involved remains to be unraveled. Widespread application of oxicams in CRC chemoprevention, as well as NSAIDs in general, is hampered by their substantial gastrointestinal toxicity [5,17]. However, novel oxicam analogues with reduced cytotoxicity have recently been synthesized [18] and proved to be efficient modulators of molecular pathways associated with CRC [19].

Heat shock proteins (HSP), including HSP70 and HSP90 families, are ubiquitous and phylogenetically conserved stress-response molecules primarily involved in the maintenance of proteostasis. Their client proteins [20,21] include numerous molecules crucial for acquiring capabilities characterizing cancer cells as proposed by Hanahan and Weinberg [2]. Consistently, HSP70 and HSP90 are overexpressed in number of cancer types, including CRC, facilitating neoplastic transformation, ensuring cancer cell proliferation, survival and invasiveness and predicting worse outcomes in cancer patients [22,23,24,25,26,27]. However, data on their expression in colorectal polyps and association with polyp potential for malignancy are missing. HSP have evoked an interest as potential chemotherapeutic targets, the inhibition of which is likely to simultaneously interfere with multiple cancer-promoting signaling pathways and thus allowing to circumvent cancer plasticity [28,29]. HSP are mostly intracellular but Hsp70α and Hsp90α can be secreted and/or displayed at cell surface, mediating cancer cell migration and interaction with immune cells [23,28,30]. 

In view of potential relevance of Hsp70α and Hsp90α in CRC chemoprevention and lack of data on their expression in precancerous polyps, we aimed to determine the ability of piroxicam and meloxicam as well as novel oxicam analogues to modulate their expression in colorectal adenocarcinoma cell lines (Caco-2, HCT 116, and HT-29) and evaluate their expression in clinical samples of colorectal neoplasms in relation to polyp potential for malignancy.

## 2. Materials and Methods

### 2.1. Patients

Biobanked paired tissue samples (Medical Ethics Committees of Wroclaw Medical University approval #KB-247/2018 from 24 April 2018) from 50 patients with colorectal polyps, admitted to the Department of Minimally Invasive Surgery and Proctology of Wroclaw Medical University for polypectomy were analyzed in the present study. Patient-matched macroscopically normal tissue was collected from 10–15 cm from polyp. The detailed patients’ characteristics is given in Table 1.

To increase number of observations and thus increase the statistical power of analysis, hyperplastic polyps were combined with tubular adenomas as polyps with none or the lowest potential for malignancy. Polyps with dominantly villous growth pattern or polyps with high grade dysplasia (as those with the highest potential for malignancy) were, in turn, combined with three cases of adenocarcinoma in polyp.

### 2.2. Oxicams

Piroxicam (compound #6) and meloxicam (compound #7) were obtained from commercial sources: Sigma-Aldrich (St. Luis, MO, USA) and Alfa Aesar (Thermo Fisher Scientific, Waltham, MA, USA), respectively, and served as reference standards. 

Novel oxicam analogues, denoted as compounds #1–5, were synthesized as previously described [31,32,33]. In short, 1,1-dioxo-1,2-benzothiazol-3-one was condensed in dimethylformamide and in the presence of triethylamine with one of the following: 2-bromoacetophenone (in case of compound #1), 2-bromo-4′-fluoroacetophenone (in case of compounds #2, #3 and #4), or 2-bromo-4′-chloroacetophenone (in case of compound #5). The resulting condensation products were subsequently rearranged in sodium ethoxide (2.3%) to the corresponding 1,2-benzothiazine ring. The final compounds were prepared by alkylation of corresponding 1,2-benzothiazine with: 1-(3-chloropropyl)-4-phenylpiperazine (in case of compounds #1 and #2) or 1-(3-chloropropyl)-4-(2-fluorophenyl)-piperazine (in case of compound #3) or with 1-(2-chloroacetyl)-4-(2-fluorophenyl)-piperazine (in case of compounds #4 and #5). The resulting products were separated and purified by crystallization from ethanol. Compounds’ structures, presented in Table 2, were confirmed by elemental (C, H, N) and spectral analyses (1H NMR, 13C NMR, FT-IR and MS).

### 2.3. Cell Cultures

Three certified human epithelial colon cancer cell lines: Caco-2 (ATCC^®^ HTB-37™), HCT 116 (ATCC^®^ CCL-247™) and HT-29 (ATCC^®^ HTB-38™) were purchased from the American Type Culture Collection (ATCC; MD, USA). Cells were cultured in water-saturated air with 5% CO_2_ at 37°C in the CELCULTURE^®^ CCL-170B-8 CO_2_ incubator (Esco Micro Pte Ltd., Singapore) in T75 polystyrene cell culture flasks (Eppendorf, AG, Hamburg, Germany). Cultures were maintained in Dulbecco’s Modified Eagle Medium (DMEM; Gibco, Thermo Fisher Scientific, Waltham, MA, USA) with addition of 10% heat inactivated fetal bovine serum (FBS; Gibco, Thermo Fisher Scientific, Waltham, MA, USA) and 1% stabilized antibiotic antimycotic solution containing 25 µg/mL of amphotericin B, 10,000 units of penicillin/mL, 10 mg/mL of streptomycin (Sigma Aldrich, MO, USA). Culture medium was changed at least twice a week. Cells were passaged after reaching approximately 80% confluency, which was preceded by harvesting with TrypLE™ Express (Gibco, Thermo Fisher Scientific, Waltham, MA, USA). Collected cells were stained with 0.4% trypan blue and counted with Countess™ Automated Cell Counter (Invitrogen, Thermo Fisher Scientific, CA, USA).

For transcriptomic and protein analyses cells were plated at 2 × 10^5^ cells per well on 6-well cell culture treated plates (Eppendorf AG, Hamburg, Germany) and maintained for 24 h at 37 °C, 5% CO_2_, followed by treatment with: meloxicam (Alfa Aesar, Thermo Fisher Scientific), and piroxicam (Sigma-Aldrich), or novel analogues of oxicams: compounds #1-5 dissolved in dimethyl sulfoxide (up-to 0.5%; DMSO) at 5 µM (24 h and 72 h), 50 µM (24 h) and 200 µM (6 h and 24 h; for transcriptomic analysis) or 50 and 200 µM (48 h; for protein analysis) concentrations. Cells cultured with 10% FBS DMEM were used as controls. Compound #1 at 200 µM, due to its lower solubility, was dissolved in 1% DMSO. Therefore, cells treated with 1% DMSO and 10% FBS DMEM were used as respective controls. 

For RNA isolation and transcriptomic analysis, post-culture media were removed and cells were lyzed with TRIzol™ Reagent (Thermo Fisher Scientific). Collected lysates were stored at −80 °C.

For protein analysis, post-culture media were removed, cells were washed twice with phosphate-buffered saline (PBS) and scraped after addition of protease inhibitor cocktail (Complete Tablets EDTA-free; Roche Diagnostics, Mannheim, Germany) in PBS. Collected cell suspensions were kept frozen at −80 °C.

### 2.4. Analytical Methods

#### 2.4.1. Transcriptomic Analysis

Total RNA was extracted from TRIzol™ cell lysates using phenol-chloroform extraction. Isolated RNA was purified using GenElute™ Mammalian Total RNA Miniprep Kit (Merck, Darmstadt, Germany) and genomic DNA contamination was avoided by on-column incubation with DNase I (Merck). cDNA was prepared following manufacturer’s protocol from 1000 ng of RNA using iScript™ cDNA Synthesis Kit (BioRad, Hercules, CA, USA). RNA concentration was determined using NanoDrop 2000 spectrophotometer (Thermo-Fisher Scientific).

For HSP analysis in patients’ samples, previously obtained [34] and biobanked cDNA was used.

Gene expression was analyzed using CFX96 Real-Time PCR system (BioRad) and SsoFast EvaGreen^®^ Supermix (BioRad). The following cycling conditions were applied: 30 s activation at 95 °C, 5 s denaturation at 95 °C, annealing/extension for 5 s at 61 °C, 40 cycles, followed by melting step (60–95 °C with fluorescent reading every 0.5 °C). 2 µL of cDNA at 1:5 dilution and 1 µL of each 10 nM forward and reverse target-specific primers were mixed with 10 µL of SsoFast EvaGreen^®^ Supermix and filled with water up to 20 µL. Primers were synthesized by Genomed (Warsaw, Poland) using intron-spanning sequences proposed by OriGene, aviliable online: https://www.origene.com/ (assessed on 10 May 2021): 5′-ACCTTCGACGTGTCCATCCTGA-3′ (for *HSPA1* forward), 5′-TCCTCCACGAAGTGGTTCACCA-3′ (for *HSPA1* reverse), 5′-TCTGCCTCTGGTGATGAGATGG-3′ (for *HSP90AA1* forward), and 5′-CGTTCCACAAAGGCTGAGTTAGC 3′ (for *HSP90AA1* reverse).

Prior analysis, an average of technical replicates Cq was calculated. Subsequently, from geometric mean of all averaged Cq values an individual sample Cq was subtracted. Resulting ΔCq were next linearized by 2^ΔCq^ conversion and normalized to internal control: *GAPDH* in cell analysis (forward: 5′-TAGATTATTCTCTGATTTGGTCGTATTGG-3′; reverse: 5′-GCTCCTGGAAGATGGTGATGG-3′) and *RN18S1* in polyp analysis (forward: 5′-ACCCGTTGAACCCCATTCGTGA-3′; reverse: 5′-GCCTCACTAAACCATCCAATCGG-3′). Resulting normalized relative quantity (NRQ) [35] were used in statistical analysis. 

#### 2.4.2. Protein Analysis

Thawed cell suspensions were sonicated on ice with the ultrasonic processor UP 200 (Hielscher Ultrasound Technology, Teltow, Germany), during two 30 sec cycles with amplitude set at 40%. To remove debris, the suspension was centrifuged (12,500× *g*, 4 °C, 10 min). Total protein concentration in the supernatants was measured colorimetrically at λ = 562 using the bicinchoninic acid assay (Thermo Fisher Scientific, Walthan, MA, USA) with Infinite^®^ M200 plate spectrophotometer (Tecan Group Ltd., Männedorf, Switzerland). Protein samples (10 µg) were diluted 1:1 with 2× Laemmli sample buffer (Bio-Rad, Hercules, CA, USA) containing 5% 2-mercaptoethanol, denatured (5 min at 95 °C), and resolved by SDS-PAGE electrophoresis. Gels (0.75 mm, 4% stacking gel and 10% resolving gel) were run using Mini-Protean Tetra Cell (Bio-Rad) at constant voltage (150 V). Subsequently, the separated proteins were transferred (30 min at constant voltage of 25 V) using Trans-Blot Turbo transfer system (Bio-Rad) to the Immobilon P membrane (Merck-Millipore, Burlington, MA, USA). Dedicated transfer buffer (25 mM Tris, 190 mM glycine, 20% methanol) was used. Membranes were blocked with 1% casein blocking buffer (Sigma-Aldrich, Saint Louis, MO, USA) at room temperature for 1 h. After washing, membranes were incubated (overnight, at 4 °C) with specific primary antibodies (R&D Systems, Minneapolis, MN, USA). The following concentrations were applied: 0.2 µg/mL HSPA1 antibodies (cat. no. MAB1663) and 0.5 µg/mL HSP90AA1/Hspα90 (cat. no. AF7247). Antibodies were diluted in pH 7.3 PBS-T buffer consisting of PBS (VWR International, Radmor, PA, USA) and 0.05% Tween-20 (Thermo Fisher Scientific, Waltham, MA, USA). Unbound antibodies were removed by washing with PBS-T (3 times, 5 min). Subsequently, membranes were incubated with 0.2 µg/mL HRP-conjugated goat anti-rabbit antibodies (Invitrogen, Carlsbad, CA, USA; cat. no. A27036) or 0.05 µg/mL HRP-conjugated goat anti-mouse antibodies (Dako A/S, Glostrup, Denmark, cat. no. P044701-2). Analyzed proteins were visualized using Clarity Western ECL chemiluminescent substrate (Bio-Rad) and ChemiDoc MP Imaging System (Bio-Rad). After visualization, probed membranes were stripped with the buffer (200 mM glycine, 0.1% SDS, 1% Tween-20, pH 2.2) and washed with PBS. Subsequently, membranes were stained for total protein with Pierce™ Reversible Protein Stain Kit for PVDF Membranes (Thermo Fisher Scientific) according to manufacturer’s instructions. The obtained Western-blot membrane images were analyzed using Image Lab software version 6.0.1 build 34 (Bio-Rad). Chemiluminescence signal intensity was normalized to signal intensity of total protein recorded for individual lanes.

### 2.5. Data Analysis

All statistical analyses were conducted using MedCalc^®^ Statistical Software version 20.008 (MedCalc Software Ltd., Ostend, Belgium; https://www.medcalc.org; 1 January 2021). Applied testes were two-sided with statistical significance set at *p* < 0.05.

Data were tested for normality with Kolmogorov-Smirnov test and for variance homogeneity with Levene test. Expression data were log-transformed prior analysis. The *t*-test for paired observations was used to analyze patient-matched samples and oxicam treated and untreated cells. Unpaired analysis was conducted using *t*-test or one-way analysis of variance (ANOVA) with Students-Neuman-Keuls *post hoc* test. For data sets with non-homogeneous variances, the Kruskal-Wallis *H* test with Conover *post hoc* test was applied. Data on protein expression were compared using *t*-test for one mean.

## 3. Results

### 3.1. HSPA1 and HSP90AA1 Expression in Patients with Colorectal Polyps

Pairwise analysis of patient-matched polyp and adjacent (normal) tissue showed a significant three-fold upregulation of both *HSPA1* and *HSP90AA1* expression in polyps (Table 3).

To identify characteristics associated with the upregulation of *HSPA1* and *HSP90AA1* expression, the fold change (polyp-to-normal) as well as gene expression in polyps and polyp-adjacent macroscopically normal tissue was referred to polyp location, histological type, dysplasia grade, size, and number.

**Figure 1:**
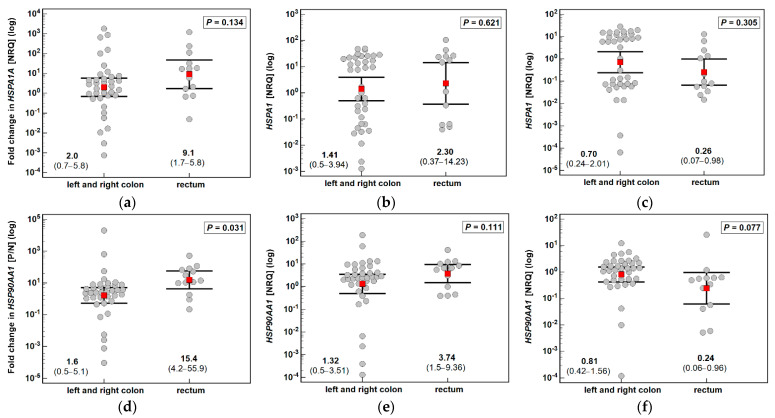
Association of HSP expression with polyp location: (**a**) fold change in *HSPA1* expression; (**b**) *HSPA1* expression in polyps; (**c**) *HSPA1* expression in polyp-adjacent (normal) tissue; (**d**) fold change in *HSP90AA1* expression; (**e**) *HSP90AA1* expression in polyps; (**f**) *HSP90AA1* expression in polyp-adjacent (normal) tissue. Data presented on logarithmic scale as geometric means (red close squares) with 95% confidence interval (whiskers) and analyzed with *t*-test for independent samples. P/N, polyp-to-normal ratio; NRQ, normalized relative quantity.

Fold change in *HSP90AA1* (by 9.4-fold) but not *HSPA1* expression was significantly greater in neoplasms located in the rectum as compared to right/left colon. It was associated with a tendency towards higher *HSP90AA1* expression in rectal polyps accompanied by lower expression in rectal polyp-adjacent mucosa (Figure 1).

**Figure 2:**
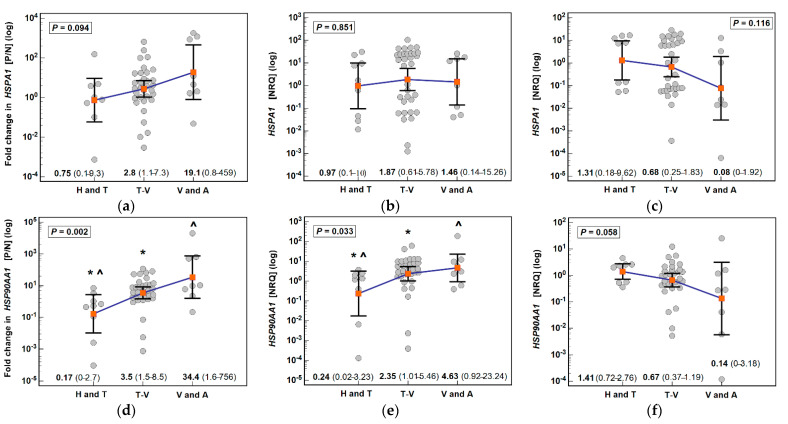
Association of HSP expression with polyp type: (**a**) fold change in *HSPA1* expression; (**b**) *HSPA1* expression in polyps; (**c**) *HSPA1* expression in polyp-adjacent (normal) tissue; (**d**) fold change in *HSP90AA1* expression; (**e**) *HSP90AA1* expression in polyps; (**f**) *HSP90AA1* expression in polyp-adjacent (normal) tissue. Data presented on logarithmic scale as geometric means (red close squares) with 95% confidence interval (whiskers) and analyzed with one-way ANOVA. Groups differing significantly (*p* < 0.05) in a *post-hoc* analysis (Students-Neuman-Keuls test) were indicated by symbols of the same type (* or ^). P/N, polyp-to-normal ratio; NRQ, normalized relative quantity; H, hyperplastic polyps; T, tubular adenomas; T-V, tubulo-villous adenomas; V, villous adenomas; A, adenocarcinomas in polyp.

Fold change in *HSPA1* (*ρ* = 0.29, *p* = 0.042) and *HSP90AA1* (*ρ* = 0.47, *p* < 0.001) expression depended on polyp type as it gradually increased along with increasing potential for malignancy. For *HSPA1*, there was no significant difference in mean fold change of expression between groups. *HSPA1* was expressed in polyps at comparable level but its expression in adjacent tissue tended to decrease along with increasing potential for malignancy (*ρ* = −0.27, *p* = 0.059) (Figure 2).

Regrading *HSP90AA1*, polyps with the lowest potential for malignancy, hyperplastic polyps and tubular adenomas, had significantly lower polyp-to normal expression ratio than polyps with polyps with higher potential for malignancy‒tubulo-villous adenomas and villous adenomas/adenocarcinomas (*ρ* = 0.47, *p* < 0.001). It was associated with changes in gene expression in both polyps and adjacent tissue. In polyps, *HSP90AA1* expression increased along with potential for malignancy (*ρ* = 0.34, *p* = 0.015) while in adjacent tissue—it decreased (*ρ* = −0.30, *p* = 0.035) (Figure 2).

**Figure 3:**
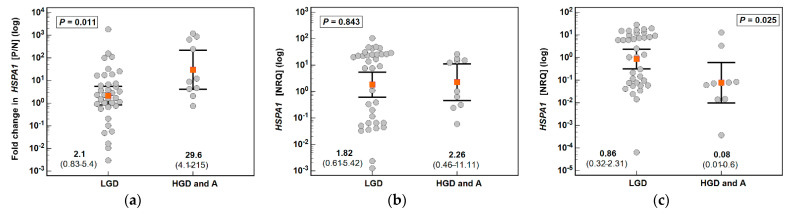

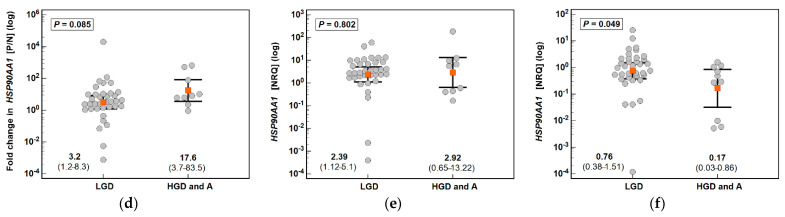
Association of HSP expression with polyp grade of dysplasia: (**a**) fold change in *HSPA1* expression; (**b**) *HSPA1* expression in polyps; (**c**) *HSPA1* expression in polyp-adjacent (normal) tissue; (**d**) fold change in *HSP90AA1* expression; (**e**) *HSP90AA1* expression in polyps; (**f**) *HSP90AA1* expression in polyp-adjacent (normal) tissue. Data presented on logarithmic scale as geometric means (red close squares) with 95% confidence interval (whiskers) and analyzed with *t*-test for independent samples. P/N, polyp-to-normal ratio; NRQ, normalized relative quantity; LGD, low grade dysplasia; HGD, high grade dysplasia; A, adenocarcinoma in polyp.

Fold change in *HSPA1* expression between polyp and normal tissue was significantly greater in patients with high grade dysplasia or adenocarcinomas in polyps. It was associated with lower gene expression in their polyp-adjacent tissue while the expression in polyp did not differ depending on dysplasia grade (Figure 3).

Likewise, fold change in *HSP90AA1* expression tended to be greater in patients with high grade polyps or adenocarcinomas due to significantly lower expression in normal polyp-adjacent mucosa (Figure 3).

**Figure 4:**
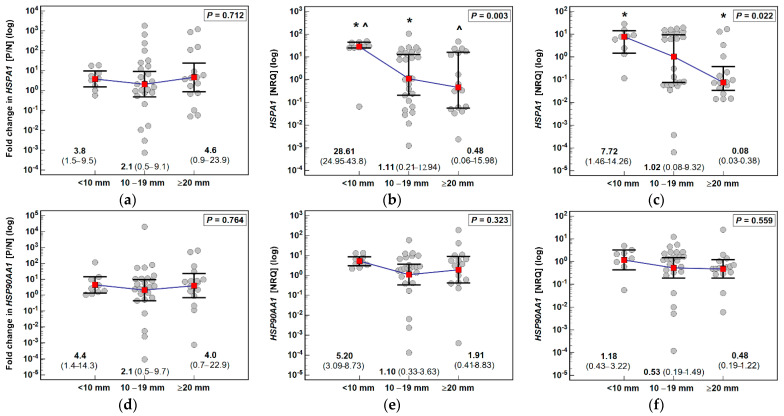
Association of HSP expression with polyp size: (**a**) fold change in *HSPA1* expression; (**b**) *HSPA1* expression in polyps; (**c**) *HSPA1* expression in polyp-adjacent (normal) tissue; (**d**) fold change in *HSP90AA1* expression; (**e**) *HSP90AA1* expression in polyps; (**f**) *HSP90AA1* expression in polyp-adjacent (normal) tissue. Data presented on logarithmic scale as geometric means (medians for *HSPA1* in polyp and adjacent mucosa) (red close squares) with 95% confidence interval (whiskers) and analyzed with one-way ANOVA (Kruskal-Wallis *H*-test). Groups differing significantly (*p* < 0.05) in a *post-hoc* analysis (Conover test) were indicated by symbols of the same type (* or ^). P/N, polyp-to-normal ratio; NRQ, normalized relative quantity.

Neither *HSPA1* nor *HSP90AA1* fold change differed significantly with respect to polyp size but *HSPA1* expression decreased along with increasing size, significantly so, in both polyps (*ρ* = −0.39, *p* = 0.006) and adjacent tissue (*ρ* = −0.39, *p* = 0.05). Comparison of medians indicated that gene expression in the largest polyps was significantly lower as compared to the smallest in polyps and adjacent tissue and also as compared to medium size in case of polyps (Figure 4).

Polyp-to-normal *HSPA1* expression ratio tended to be higher in patients with single than multiple polyps (5.4 vs. 0.8, *p* = 0.093) but there was no significant difference in its expression in polyp (*p* = 0.203) or normal mucosa (*p* = 0.460).

Polyp-to-normal *HSP90AA1* expression ratio did not differ significantly between patients with single than multiple polyps as well (4.5 vs. 1.2, *p* = 0.167) and there was no significant difference in its expression in polyp (*p* = 0.693) or normal mucosa (*p* = 0.101).

### 3.2. Effect of Oxicams on HSPA1 and HSP90AA1 Expression in Colorectal Adenocarcinoma Cell Lines Caco-2, HCT 116 and HT-29

#### 3.2.1. HSPA1/Hsp70α and HSP90AA1/Hsp90α Proteins

To investigate the ability of classic (piroxicam and meloxicam) and novel analogues (compounds #1–5) to modulate HSPA1 and HSP90AA1 protein expression, Caco-2, HCT 116, and HT-29 were treated with 50 and 200 μM drug concentration for 48 h and cell protein content was analyzed by Western-blotting.

**Figure 5:**
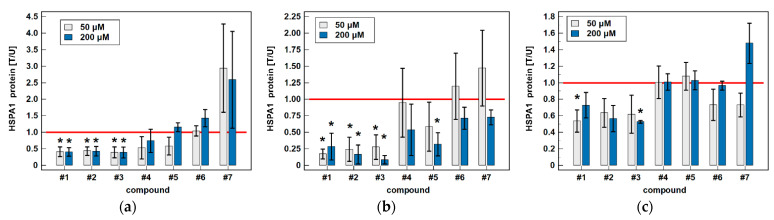
Effect of oxicams on HSPA1 protein expression in colorectal adenocarcinoma cell lines: (**a**) Caco-2; (**b**) HCT 116; (**c**) HT-29. Data presented as means ± SEM (*n* = 3) of normalized expression in treated against untreated cells [T/U] and analyzed using *t*-test for one mean. Statistical significance (*p* < 0.05) is indicated by asterisks (*) and protein expression in control (untreated) cells is indicated by horizontal red solid line. Cells were treated with 50 μM or 200 μM oxicams for 48 h. Compound #6 denotes piroxicam and #7—meloxicam.

Regarding HSPA1 and classic drugs, there was large variation in cell response and none of the observed effects was statistically significant (Figure 5).

**Figure 6:**
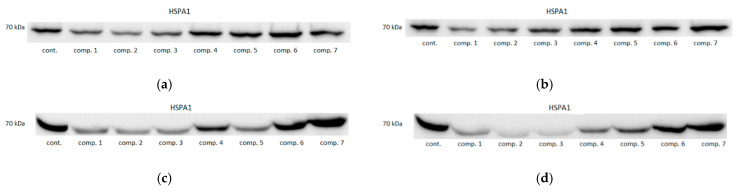

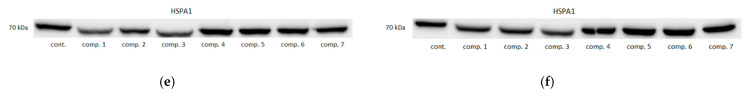
Effect of oxicams on HSPA1 protein expression in colorectal adenocarcinoma cell lines-exemplary blots: (**a**) in Caco-2 treated with 50 μM oxicams; (**b**) in Caco-2 treated with 200 μM oxicams; (**c**) in HCT 116 treated with 50 μM oxicams; (**d**) in HCT 116 treated with 200 μM oxicams; (**e**) in HT-29 treated with 50 μM oxicams; (**f**) in HT-29 treated with 200 μM oxicams. Cont., control; comp., compound.

Oxicam analogues downregulated HSPA1 protein expression, to varying degrees, in all examined cell lines (Figure 5). In Caco-2 cells, compound #1 at 50 and 200 μM concentration downregulated protein expression by 2.4- and 2.5-fold, compound #2 by 2.3- and 2.2-fold and compound #3 by 2.5- and 2.6-fold. In HCT 116 cells, compound #1 at 50 and 200 μM concentration downregulated protein expression by 5.8- and 3.5-fold, compound #2 by 4.1- and 6.0-fold, compound #3 by 3.6- and 11.5-fold, and compound #5 by 1.7-fold (non-significantly) and by 3.1-fold. In HT-29 cells, 50 μM compound #1 and 200 μM compound #3 downregulated HSPA1 by 1.9-fold (Figure 5). Representative blots are presented in Figure 6.

**Figure 7:**
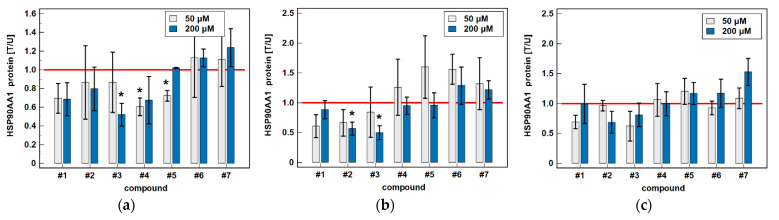
Effect of oxicams on HSP90AA1 protein expression in colorectal adenocarcinoma cell lines: (**a**) Caco-2; (**b**) HCT 116; (**c**) HT-29. Data presented as means±SEM (*n* = 3) of normalized expression in treated against untreated cells [T/U] and analyzed using *t*-test for one mean. Statistical significance (*p* < 0.05) is indicated by asterisks (*) and protein expression in control (untreated) cells is indicated by horizontal red solid line. Cells were treated with 50 μM or 200 μM oxicams for 48 h. Compound #6 denotes piroxicam and #7—meloxicam.

**Figure 8:**
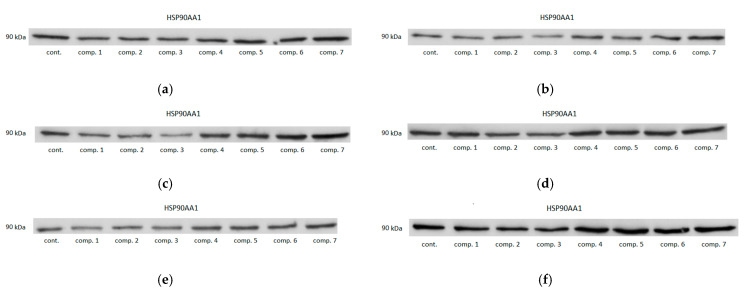
Effect of oxicams on HSP90AA1 protein expression in colorectal adenocarcinoma cell lines-exemplary blots: (**a**) in Caco-2 treated with 50 μM oxicams; (**b**) in Caco-2 treated with 200 μM oxicams; (**c**) in HCT 116 treated with 50 μM oxicams; (**d**) in HCT 116 treated with 200 μM oxicams; (**e**) in HT-29 treated with 50 μM oxicams; (**f**) in HT-29 treated with 200 μM oxicams. Cont., control; comp., compound.

None of classic oxicams had significant and consistent effect on HSP90AA1 expression. Of novel drugs, compounds #1–3 tended to be inhibitory in all cell lines and compounds #4 and #5 in Caco-2. Statistically significant effect was observed for 200 μM compound #3 in Caco-2 (by 1.9-fold) and HCT 116 (by 2-fold) and compound #2 in HCT 116 (by 1.8-fold) and for 50 μM compound #4 and #5 in Caco-2 (by 1.7- and 1.4-fold, respectively) (Figure 7). Representative blots are presented in Figure 8.

#### 3.2.2. HSPA1 and HSP90AA1 Transcripts

Transcriptional analysis was conducted on HCT 116 and Caco-2 cells as more responsive to oxicam treatment. To investigate the dose-dependent effect of oxicams on *HSPA1* and *HSP90AA1* gene expression, cells were treated with 5, 50 and 200 μM drug concentration for 24 h and gene expression was analyzed with RTqPCR. In addition, the effect of time was determined in 6 and 24-h cultures treated with 200 μM oxicams and in 24 and 72-h cultures treated with 5 μM oxicams.

**Figure 9:**
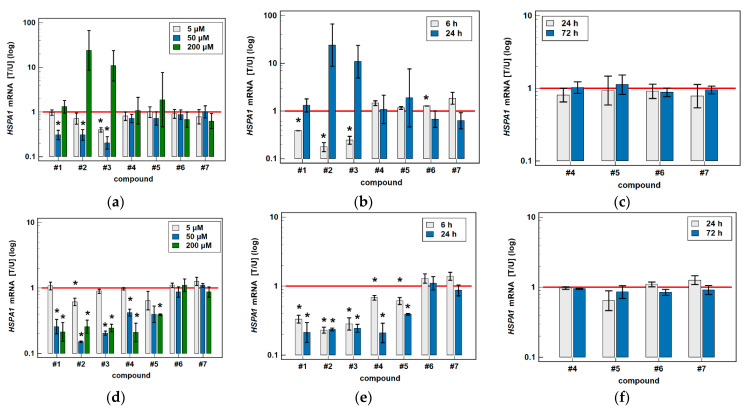
Effect of oxicams on *HSPA1* expression in colorectal adenocarcinoma cell lines: (**a**) dose-dependent effect in Caco-2 cells treated for 24 h; (**b**) time-dependent effect in Caco-2 cells treated with 200 μM oxicams; (**c**) time-dependent effect in Caco-2 cells treated with 5 μM oxicams; (**d**) dose-dependent effect in HCT 116 cells treated for 24 h; (**e**) time-dependent effect in HCT 116 cells treated with 200 μM oxicams; (**f**) time-dependent effect in HCT 116 cells treated with 5 μM oxicams. Data presented on logarithmic scale. Bars represent mean expression ratio of treated-to-untreated cells [T/U] with 95% confidence interval (whiskers). Statistically significant differences between treated and untreated cells as analyzed by *t*-test for paired samples are indicated by asterisks (*). Horizontal solid red line represents gene expression in control (untreated samples). Compound #6 denotes piroxicam and #7—meloxicam.

**Figure 10:**
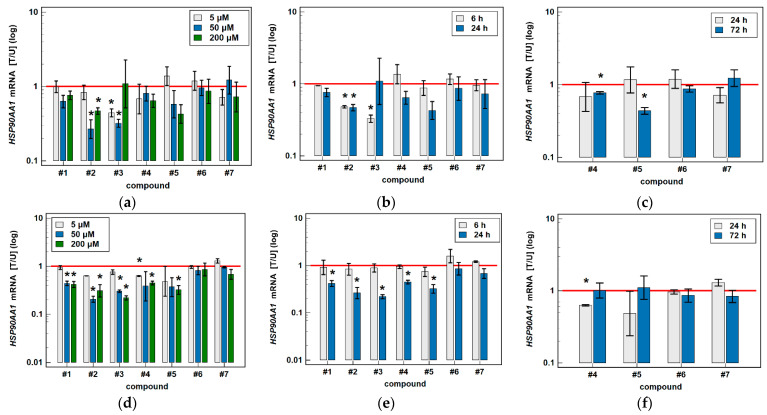
Effect of oxicams on *HSP90AA1* expression in colorectal adenocarcinoma cell lines: (**a**) dose-dependent effect in Caco-2 cells treated for 24 h; (**b**) time-dependent effect in Caco-2 cells treated with 200 μM oxicams; (**c**) time-dependent effect in Caco-2 cells treated with 5 μM oxicams; (**d**) dose-dependent effect in HCT 116 cells treated for 24 h; (**e**) time-dependent effect in HCT 116 cells treated with 200 μM oxicams; (**f**) time-dependent effect in HCT 116 cells treated with 5 μM oxicams. Data presented on logarithmic scale. Bars represent mean expression ratio of treated-to-untreated cells [T/U] with 95% confidence interval (whiskers). Statistically significant differences between treated and untreated cells as analyzed by t-test for paired samples are indicated by asterisks (*). Horizontal solid red line represents gene expression in control (untreated samples). Compound #6 denotes piroxicam and #7—meloxicam.

Classic oxicams had negligible/non-significant effect on *HSPA1* (Figure 9) and *HSP90AA1* expression (Figure 10).

*HSPA1* was downregulated by oxicam analogues #1–3 in Caco-2 and #1–5 in HCT-116 cells. Compound #1 at 50 μM concentration in 24-h cultures or at 200 μM concentration in 6-h cultures downregulated *HSPA1* by 3.3- and 2.5-fold in Caco-2 and compound #2 by 3.3- and 5.7-fold, respectively. Compound #3 downregulated *HSPA1* at 5 and 50 μM concentration (by 2.5- and 4.9-fold) in 24-h cultures and at 200 μM in 6-h cultures (by 4.1-fold). HCT 116 cells were more sensitive to novel oxicams—compound #1 downregulated *HSPA1* in 24-h cultures at 50 and 200 μM (by 2.8- and 4.7-fold) and in 6-h cultures (by 3.0-fold). Compound #2 downregulated *HSPA1* in 24-h cultures at 5, 50 and 200 μM (by 1.6-, 6.8- and 4.2-fold) and in 6-h cultures (by 4.3-fold). Compound #3 downregulated *HSPA1* in 24-h cultures at 50 and 200 μM (by 4.9- and 4.1-fold) and in 6-h cultures (by 3.5-fold) and compound #4 by 2.4-, 4.8- and 1.5-fold, respectively. Compound #5 was effective at 200 μM concentration in 6 and 24-h cultures (downregulation by 1.6- and 2.6-fold) (Figure 9).

Oxicams downregulated expression of *HSP90AA1* gene more markedly than protein: compounds #2–5 in Caco-2 cells and compounds #1–5 in HCT 116 cells. Compound #2 in Caco-2 downregulated gene expression by 3.7- and 2.1-fold at 50 and 200 μM in 24-h cultures and by 2.1-fold at 200 μM in 6-h cultures. Compound #3 downregulated *HSP90AA1* by 2.3- and 3.1-fold at 5 and 50 μM in 24-h cultures and by 3.0-fold at 200 μM in 6-h cultures. The effect of compounds #4 and #5 was non-significant/negligible in 6 and 24-h cultures but 72-h incubation with low (5 μM) drug concentration downregulated gene expression by 1.3- and 2.3-fold, respectively.

In more susceptible HCT 116 cells, compound #1 downregulated *HSP90AA1* at 50 and 200 μM in 24-h cultures by 2.3- and 2.4-fold, compound #2 by 5.0- and 3.8-fold, and compound #3 by 3.3 and 4.5-fold. The effect of compound #4 was significant for treatment with 5 and 200 μM (by 1.6- and 2.2-fold) and for compound #5 for treatment with 200 μM (by 3.1-fold) (Figure 10).

## 4. Discussion

Discerning the molecular mechanisms underlying drugs’ anti-tumor activity and detailed understanding of expression patterns and relevance of prospective targets is prerequisite for developing safe and effective molecular therapies. It is also crucial in enabling design of chemicals with improved characteristics [36]. Owing to their central role in proteostasis and cell signaling, heat shock proteins constitute a unique target for antineoplastic therapies holding promise to circumvent cancer plasticity [28,29]. Here, we showed that both *HSPA1* and *HSP90AA1* transcripts are upregulated, to the same degree, in colorectal polyps as compared to polyp-adjacent tissue. 

HSPA1/Hsp70 is a prototypical and an inducible member of HSP70 family, which guards cancer cells against stress-induced proteotoxicity. Consistently, it is overexpressed in number of cancers, although clinical data regarding CRC are, as observed by Gao at al. [37], surprisingly scanty. Lazaris et al. [38] showed 77% of colorectal tumor samples to contain at least 10% of neoplastic cells with Hsp70-immunoreactivity. The immunoreactivity was positively correlated with cancer aggressiveness, as it increased along with tumor dedifferentiation, and inversely with patients’ survival. The association between Hsp70 protein expression and patients’ prognosis, but not tumor grade or cancer stage, has subsequently been confirmed by others [39,40,41]. Likewise, Hsp70 elevation in serum has been linked with poor prognosis and the disease advancement [24]. DLD-1 cells with downregulated *HSPA1* expression responded to a treatment with a heat shock response-inducing agent with a 3-fold upregulation [42]—which corresponds with a degree of average elevation in gene expression in polyps observed in clinical samples evaluated in current study. Mechanistically, *HSPA1*/Hsp70 expression improved migratory properties of DLD-1 cells and its knockdown reduced by several-fold the expression of Snail, Snug and Twist, the E-cadherin suppressors and inductors of epithelial-mesenchymal transition [42]. Others have shown selective *HSPA1* knockout to enhance cancer cell immunogenicity [43] and induce cell death in xenografts independent from caspase/Bcl-2 pathway [44].

To the best of our knowledge, *HSPA1* expression in precancerous colorectal lesions has not been addressed. Herein, *HSPA1* expression rate between polyp and adjacent tissue increased significantly along with increasing potential for malignancy. Interestingly, however, the rising trend resulted from diminishing expression in histologically normal polyp-adjacent tissue while polyp expression remined comparable in hyperplastic/tubular polyps with low potential for malignancy, tubulo-villous with medium potential for malignancy and villous polyps/adenocarcinomas in polyps with the highest potential for malignancy. Likewise, *HSPA1* expression rate was significantly higher in polyps with high grade dysplasia, again owing to a drop in the gene expression in adjacent tissue. Of note, alterations in molecular landscape of histologically and morphologically normal tissue surrounding colorectal adenocarcinomas have repeatedly been demonstrated [19,34,45] and suspected of contributing to synchronous tumors and/or cancer recurrence after curative resection [46]. Apart from polyp type and dysplasia grade, malignant potential is believed to correlate directly with polyp size [47]. The rate of *HSPA1* expression was not associated with polyp size in the examined cohort. However, unexpectedly, our results showed that gene expression decreased alongside increasing polyp size, both in polyp and polyp-adjacent tissue, which might imply a protective role for *HSPA1*/Hsp70 prior neoplastic transformation. Low level of gene expression may increase susceptibility of cellular protein to stress-induced damage and thus facilitate transformation. However, contradicting the notion, transforming potential of *HSPA1*/Hsp70 overexpression, and not downregulation, has been demonstrated in Apc^Min/+^ mouse model of CRC. The model is characterized by development of adenomas in the small and large intestine. Tao et al. [41] showed that the loss of Hsp70 reduces the number and size of adenomas and decreases their proliferation rate and resistance to apoptosis as compared to Hsp70-expressing mice. Mechanistically, loss of Hsp70 attributed to enhanced degradation of β-catenin without an effect on its gene expression while Hsp70 expression activated of Akt, ERK, and p38/MAPK pathways [41]. Likewise, *HSPA1*/Hsp70 has been necessary for neoplastic transformation of mammary epithelial cells induced by Her2 oncogene [48]. Taken together, in vitro and animal-based findings evoked an interest in *HSPA1*/Hsp70 as potential target for chemoprevention in addition to anti-tumor therapies.

Therefore, we evaluated the ability of classic, piroxicam and meloxicam, as well as novel oxicam analogues to modulate HSPA1/Hsp70 expression in HCT 116, Caco-2 and HT-29 cells. Regarding classic oxicams, neither protein nor mRNA expression were significantly affected as there was rather high variability in cell response between biological replicates. Unlike classic oxicams, their novel analogues were effective in downregulating HSPA1 already at 50 μM concentration at protein and mRNA level. HCT 116 cells were the most sensitive to oxicam analogues and the line has been claimed to not express COX2 [49]. Therefore, it is likely that HSP-inhibiting effect is not mediated by drug ability to inhibit enzyme activity.

Structurally, all tested analogues differ from classic drugs with arylpiperazine pharmacophore and benzoyl moiety substitutions at thiazine ring. It has previously been noted that such modification enhances anti-inflammatory properties of the drug, owing to high electron-withdrawing properties of arylpiperizine pharmacophore [50]. Indeed, the presence of this moiety has allowed novel analogues to regulate expression of enzymes involved in L-arginine/nitric oxide pathway [19] as well as monocyte/macrophage-associated chemokines (manuscript submitted). Like in case of L-arginine/nitric oxide pathway enzymes and chemokines, compounds with 3-carbon propylene linker between nitrogen atoms of thiazine and piperazine rings (compounds #1, #2 and #3) were generally more effective in downregulating HSPA1 than those with 2-carbon oxyethylene linker (compounds #4 and #5). Its presence was crucial for gene downregulation in Caco-2 cells. Of the evaluated analogues, compounds #2 and #3 seem to be the most effective, consistently downregulating both HSPA1 protein and mRNA in both cell lines, with compound #3 significantly downregulating HSPA1 also in HT-29 cells. They are distinguished from compound #1 by the presence of fluoro-substituents at arylpiperazine ring, likely to further enhance the electron-withdrawing and thus anti-inflammatory properties of the pharmacophore. Of note, as HSPs may engage receptors involved in NFκB activation [27], some anti-inflammatory effects exerted by novel oxicams containing arylpiperazine pharmacophore, but not classic drugs from this group, e.g., chemokine downregulation, might be mediated by their inhibitory effect on HSPA1. Intriguingly, a biphasic type of response was observed in case of *HSPA1* mRNA expression in Caco-2 cells, previously noted also in case of L-arginine/nitric oxide pathway enzymes [19] and chemokines (manuscript submitted). The gene downregulation after 24-h incubation with lower analogue concentration (5 and/or 50 μM) or 6-h incubation with 200 μM but its upregulation following 24-h incubation with 200 μM drugs.

Tested oxicam analogues, but not classic drugs, were effective also in downregulating expression of *HSP90AA1*. Like for *HSPA1*, HCT 116 cells were more responsive and compounds #2 and #3 more efficient than other ones, but the effect seemed to be generally slightly less marked. The inhibition was less evident at protein level. HSP90AA1 protein was significantly decreased upon treatment of Caco-2 with compounds #3–5 and HCT 116 with compounds #2 and #3. Regarding clinical samples, *HSP90AA1* was upregulated in polyps to the very same degree as *HSPA1.* Like *HSPA1*, its expression ratio (polyp-to-normal) was dependent on polyp malignancy potential. It was higher in polyps with dominant villous growth pattern and high grade of dysplasia. In case of dysplasia grade, the expression pattern resembled that of *HSPA1*: the higher expression ratio resulted from lower gene expression in adjacent tissue while polyp expression did not differ between polyps with low and high dysplasia grade. In case of polyp type, the *HSP90AA1* expression rate increasing along with growing contribution of villous growth pattern resulted not from both decreasing gene expression in adjacent tissue and from increasing expression in polyp. Unlike *HSPA1*, *HSP90AA1* expression was not associated with polyp size but was higher in case of rectal than colonic polyps.

Neoplastic transformation is believed to occur as a result of overwhelming protective Hsp90 capacity during periods of high cellular stress [23]. In this respect, diminishing *HSP90AA1* expression in normal polyp-adjacent tissue along with increasing polyp potential for malignancy renders cellular proteins susceptible to stressors, which may translate into creating tumor-promoting environment. In transformed cells, Hsp90 acts to preserve malignant phenotype by facilitating accumulation of beneficial while suppressing mutations lethal for cancer cells [23]. Therefore, higher *HSP90AA1* expression in polyps with greater potential for malignancy, observed in evaluated clinical samples, agrees well with Hsp90 function as a key facilitator of unrestrained growth owned to Hsp90-mediated stabilization of proteins involved in proliferation [23]. In the light of significance of this heat shock protein for cancer adaptability to endogenous (e.g., oxidative and metabolic stress, hypoxia) and exogeneous (e.g., chemo/radiotherapy) stressors as well as its role in stabilization of oncogenic proteins, particularly those facilitating cancer growth, invasion and metastasis [23,51], markedly weaker effect of oxicam analogues on HSP90AA1 protein is disappointing. Targeting Hsp90 in CRC is of particular interest due to high incidence of *KRAS* mutations and constitutive activation of Ras/Raf/MEK/Erk signaling, pathways susceptible to Hsp90 inhibition. Moreover, Hsp90 inhibition sensitizes colorectal cancer cells to oxaliplatin and the underlying molecular mechanisms involves hampering NFκB signaling [52]. The ability of investigated oxicams to downregulate gene but not protein expression might imply an involvement of counteractive posttranslational mechanisms, which warrants further investigation.

## 5. Conclusions

The expression of *HSPA1* and *HSP90AA1*, key heat shock proteins involved in facilitating neoplastic transformation and cancer development, is altered already in precancerous colorectal lesions and surrounding tissue, to degree dependent on polyp potential for malignancy. In colorectal cancer cells, the number of *HSPA1* and *HSP90AA1* transcripts as well as the amount of HSPA1 and, to lesser degree, HSP90AA1 protein can be altered by novel oxicam analogues containing arylpiperazine pharmacophore and benzoyl moiety substitutions at thiazine ring instead of methyl substituent at position 2 and 2-peridocarbamoyl substituent at position 3, respectively. Analogue efficacy was dependent on the presence of 3-carbon propylene linker between thiazine and piperazine nitrogens and on fluorine substituents at arylpiperazine pharmacophore.

## Figures and Tables

**Table 1:** Characteristics of patients with colorectal polyps.

Parameter	Characteristics
*N*	50
Sex distribution [F/M], *n*	23/27
Age [yrs.], mean (95%CI)	62.9 (59.1–66.7)
Polyp histology, *n*:	
tubular adenoma	5
tubulo-villous adenoma	33
villous adenoma	5
hyperplastic polyps	4
adenocarcinomas	3
Grade of dysplasia, *n*:	
low	10
high	36
Polyp size, *n*:	
<10 mm	9
10–19 mm	25
≥20 mm	16
Polyp location, *n*:	
left colon	22
right colon	15
rectum	13
Number of polyps:	
One	34
Multiple	26

*N*, number of observations; F/M, female-to-male ratio; yrs., years; CI, confidence interval.

**Table 2:** Chemical structures of examined oxicams.

Oxicam	Structure
Compound #**1**	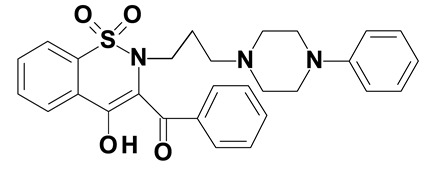
Compound #**2**	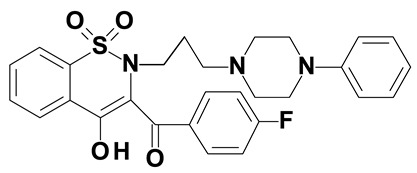
Compound #**3**	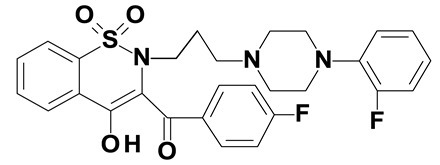
Compound #**4**	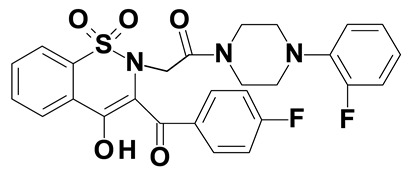
Compound #**5**	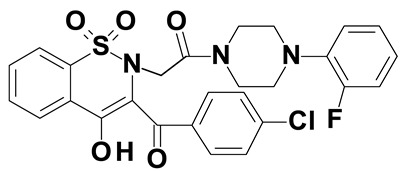
Piroxicam #**6**	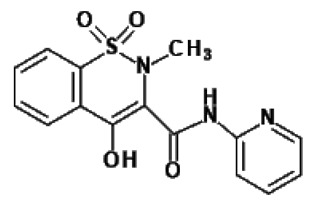
Meloxicam #**7**	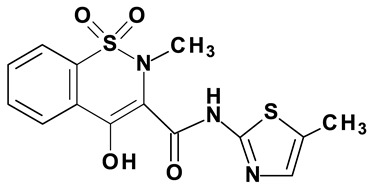

**Table 3:** Heat shock protein *HSPA1* and *HSP90AA1* expression in patients with colorectal polyps.

Gene	Mean Expression (95%*CI*) [NRQ]		FC [P/N], *p*
	Normal Mucosa	Polyp	
*HSPA1*	0.54 (0.23‒1.27)	1.60 (0.67‒3.80)	2.97, *p* = 0.016
*HSP90AA1*	0.59 (0.32‒1.07)	1.73 (0.82‒3.69)	2.94, *p* = 0.023

*CI*, confidence interval; NRQ, normalized relative quantity; FC, fold change; P/N, polyp-to normal ratio. Data analyzed using *t*-test for paired samples.

## Data Availability

Not applicable.
